# ChatGPT: A Supplemental Tool for Efficiency and Improved Communication in Rural Dermatology

**DOI:** 10.7759/cureus.43812

**Published:** 2023-08-20

**Authors:** Mindy N Baker, Clayton P Burruss, Chase L Wilson

**Affiliations:** 1 Dermatology, University of Kentucky College of Medicine, Lexington, USA; 2 Dermatology, Elkhorn Dermatology, Georgetown, USA

**Keywords:** rural healthcare access, rural dermatology, quality of care, language models, artificial intelligence, machine learning

## Abstract

In November 2022, OpenAI released version 3.5 of ChatGPT, the first publicly available artificial intelligence (AI) language model designed to engage in natural, human-like dialogue with users. While this groundbreaking technology has been extensively studied in various domains, its potential applications in rural dermatology remain unexplored in the existing literature. Our research investigates the many benefits that ChatGPT could offer in rural dermatology, particularly concerning administrative tasks and communication with communities with lower healthcare literacy. However, we also acknowledge that utilizing ChatGPT without proper caution and discernment may lead to potential drawbacks. This paper examines the opportunities and challenges associated with integrating ChatGPT into rural dermatology practices, ultimately fostering a well-informed and responsible approach to its implementation.

## Editorial

In rural settings, dermatologists often face unique challenges that hinder the delivery of optimal care, primarily due to the presence of healthcare disparities. Common barriers include limited access to specialized care, inadequate resources, and lower healthcare literacy [1]. Additionally, dermatologists in rural areas often bear significant administrative responsibilities, which can take the focus away from direct patient care.

**Figure 1:**
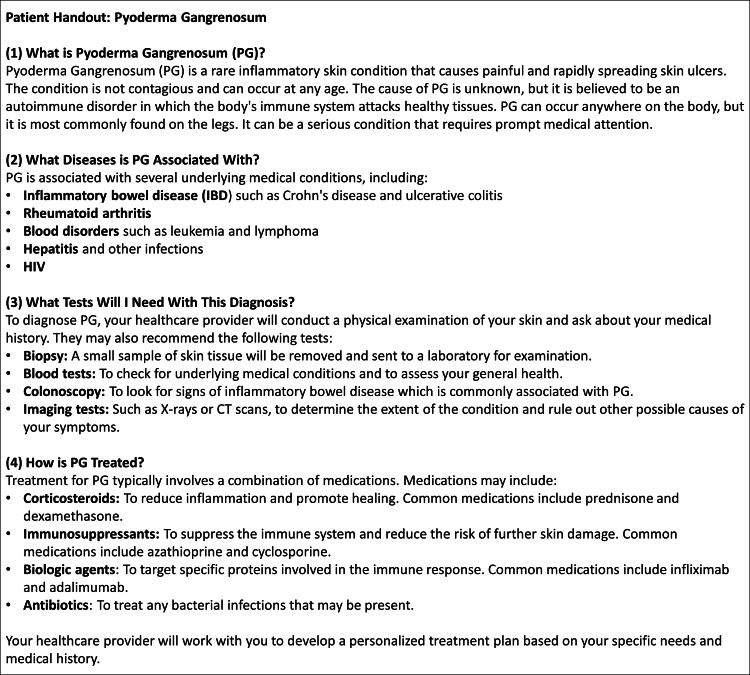
ChatGPT output demonstrating an iterative patient educational handout on pyoderma gangrenosum. Text generated by ChatGPT on February 20, 2023.

Addressing these disparities and streamlining administrative tasks is crucial to improving healthcare outcomes in rural dermatology. ChatGPT, an accessible artificial intelligence (AI) language model, can assist dermatologists in overcoming these barriers. We discovered that ChatGPT can be used to generate disease-specific educational patient handouts for common dermatological conditions (Figure 1). The adaptability of ChatGPT allows for the modification of factors such as document length, word choice, and language [2]. This also allows handouts to be customized to patients’ various education levels, cultures, and ages. In our study, we used ChatGPT to create patient handouts through an iterative process. The specificity of the initial input significantly influenced the number of iterations required to refine the output. Inputs with more specifications underwent fewer iterations, resulting in a more precise and targeted educational resource. On average, we found that three to 10 iterations were necessary per handout, with fewer iterations needed for more specific initial inputs.

The potential impact of ChatGPT in rural dermatology is vast. By automating the creation of educational materials, ChatGPT can aid dermatologists in efficiently communicating with patients and communities with lower healthcare literacy levels. Even though our study only includes ChatGPT’s output for a pyoderma gangrenosum handout, educational healthcare material can be generated for most dermatologic conditions. A powerful advantage of utilizing ChatGPT as a supplement is how content can easily be adjusted for each patient in mere seconds. Prompting ChatGPT to write with simple language and practical advice for rural patients can provide better understanding and adherence to treatment plans, ultimately improving patient outcomes.

**Figure 2:**
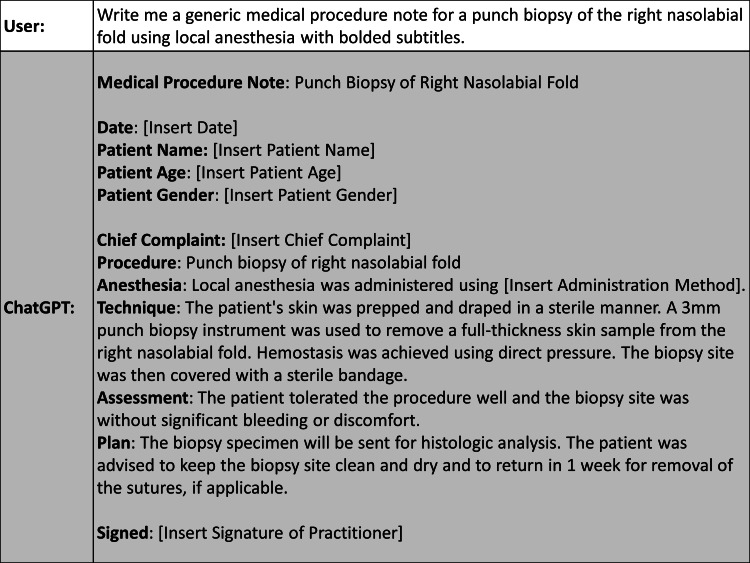
ChatGPT output demonstrating an AI-generated procedural note template. Text generated by ChatGPT on February 6, 2023. AI: artificial intelligence.

Moreover, we found ChatGPT’s abilities can extend to assisting with administrative tasks. This is seen with our generation of procedural note templates and drafts of prior authorization requests (Figure 2). While our example shows how a medical procedure note could be generated for a punch biopsy on the right nasolabial fold, the possibilities do not stop here. ChatGPT’s outputs can be customized to a multitude of procedures for other body locations in mere seconds as well. As a result, this can significantly reduce the administrative burden for rural dermatologists who may lack robust electronic medical records with pre-formed templates. This streamlined approach has the potential to enhance overall practice efficiency and enable dermatologists to devote more time and focus to direct patient care.

However, we must recognize the need for caution, proofreading, and verification when adopting ChatGPT. Limitations of ChatGPT to dermatology include its text-based interface and its limited training data which extends to 2021 [3]. There is also the risk of automation bias [4], whereby clinicians may unquestioningly follow the AI-generated guidance without critical evaluation. In medicine, where information is constantly evolving, ChatGPT may not provide the most up-to-date medical advice for dermatology patients. Additionally, dermatology is a complex specialty where a thorough medical history of a patient often determines a patient’s treatment plan. ChatGPT may not take all patient factors into account, while a human dermatologist would. Finally, if AI software is trained on information that is biased, then outputs may reflect these biases as well. Expert supervision is, therefore, essential to guarantee output validity. Despite the clinical and ethical concerns regarding AI in healthcare [5], ChatGPT has the potential to improve patient care and increase healthcare efficiency in a rural dermatology setting.

In conclusion, ChatGPT presents as a promising tool to address the rural disparities in dermatology by mitigating healthcare literacy challenges and simplifying administrative tasks. Utilizing AI to generate medical handouts and templates specific to rural dermatology has proven to be considerably faster than writing from tabula rasa, even if multiple adjustments need to be made. As we further study the applications of AI in healthcare, thoughtful and responsible integration of ChatGPT can pave the way for reduced dermatologic disparities in rural populations.
